# Dr. Chauncey W. Chidester

**Published:** 1917-12

**Authors:** 


**Dr. Chauncey W. Chidester,** P. & S. Chicago, 1886, died at his home in Newark Valley, Aug. 16, of arterio-sclerosis, aged 70. He was formerly coroner of Tioga Co.

